# Medical Compliance of Fibrate and the Decreased Risk of Age-Related Macular Degeneration in Dyslipidemia-Related Diseases: A Population-Based Cohort Study

**DOI:** 10.3390/ijerph18010301

**Published:** 2021-01-03

**Authors:** Kai Wang, Ming-Ju Hsieh, Hsiang-Wen Chien, Chia-Yi Lee, Chao-Bin Yeh, Jing-Yang Huang, Shun-Fa Yang

**Affiliations:** 1 Department of Ophthalmology, Cathay General Hospital, Taipei 106, Taiwan; cgh04979@cgh.org.tw (K.W.); bmw35chien1@gmail.com (H.-W.C.); 2 Department of Ophthalmology, Sijhih Cathay General Hospital, New Taipei City 211, Taiwan; 3 School of Medicine, College of Medicine, Fu Jen Catholic University, New Taipei 242, Taiwan; 4 Institute of Medicine, Chung Shan Medical University, Taichung 402, Taiwan; 170780@cch.org.tw (M.-J.H.); wchinyang@gmail.com (J.-Y.H.); 5 Oral Cancer Research Center, Changhua Christian Hospital, Changhua 500, Taiwan; 6 Graduate Institute of Biomedical Sciences, China Medical University, Taichung 404, Taiwan; 7 Department of Ophthalmology, Show Chwan Memorial Hospital, Changhua 500, Taiwan; ao6u.3msn@hotmail.com; 8 Department of Emergency Medicine, School of Medicine, Chung Shan Medical University, Taichung 402, Taiwan; sky5ff@gmail.com; 9 Department of Emergency Medicine, Chung Shan Medical University Hospital, Taichung 402, Taiwan; 10 Department of Medical Research, Chung Shan Medical University Hospital, Taichung 402, Taiwan

**Keywords:** age-related macular degeneration, epidemiology, fibrate, dyslipidemia, compliance

## Abstract

The purpose of the current study is to evaluate the incidence of age-related macular degeneration (AMD) in dyslipidemia-related diseases with or without the use of fibrate. Patients were defined as dyslipidemia-related diseases according to the diagnostic code and lab exam arrangement, then the population was divided into those with fibrate application and those without via 1:2 ratios of propensity-score matching. The primary outcome is the development of AMD after dyslipidemia-related diseases by the Cox proportional hazard regression. Besides, the relationship between the medical compliance of fibrate, presented as medical possession ratio (MPR), and the AMD development was also analyzed. A total of 22,917 patients and 45,834 individuals were enrolled in the study and control groups. There were 572 and 1181 events of any AMD development in the study and control groups which showed identical risk of AMD (aHR: 0.94, 95% CI: 0.85–1.04). However, a reduced risk of any AMD was found in those patients reached a baseline MPR more than 20% (aHR: 0.729, 95% CI: 0.599–0.887, *p* = 0.0016) and overall MPR more than 5% three years after the diagnosis of dyslipidemia-related diseases (aHR: 0.712, 95% CI: 0.557–0.909, *p* = 0.0065). Besides, a lower risk of dry-AMD was also found in those patients with the above conditions (aHR: 0.736, 95% CI: 0.599–0.906, *p* = 0.0038 and aHR: 0.721, 95% CI: 0.557–0.934, *p* = 0.0133, respectively). In conclusion, the use of fibrate with fair initial medical compliance will decrease the incidence of AMD in patients with dyslipidemia-related diseases, especially for the development of dry-AMD.

## 1. Introduction

Dyslipidemia refers to the dysregulation of blood lipid level which features elevated low density lipoprotein and very low density lipoprotein or triglyceride in different subtypes [1]. Recently, the prevalence of dyslipidemia has been estimated to be above 20% in the European population and the age-standardized prevalence of treatment-need dyslipidemia in Japan is more than 30% [1,2]. Dyslipidemia is related to a numbers of atherosclerotic cardiovascular co-morbidities, including the coronary heart disease, peripheral arterial occlusive disease and cerebrovascular disease, which can lead to death [3]. Consequently, dyslipidemia treatment is necessary to prevent further mortality, in which both the statin and fibrate have been used frequently to manage the lipid dysregulation [4].

Aside from the systemic morbidities, dyslipidemia is also associated with certain ocular diseases [5,6]. Retinal vascular occlusions, which could be sight-threatening due to macular ischemia and ruberosis iridis, can occur more frequently in patients with hyperlipidemia and atherosclerotic change [7]. Also, dyslipidemia and atherosclerosis could lead to subsequent internal carotid artery stenosis and ocular ischemic syndrome [8]. On the other hand, age-related macular degeneration (AMD) which features the deposition of lipid materials between the retinal pigment epithelium and Bruch’s membrane [9], is also related to the presence of dyslipidemia [10]. In previous experience, the patients with high cholesterol as well as triglyceride levels are under greater risk of developing AMD [10,11].

As far as the relationship between dyslipidemia treatment and the occurrence of AMD is concerned, several studies have proposed a protective effect of statin against the AMD development [12,13,14]. According to a previous systemic review, the risk of early AMD was significantly reduced in patients with dyslipidemia who took statin as treatment agent [12]. Besides, the visual acuity in patients with AMD was improved after high-dose statin application [14]. Nevertheless, there is little research that surveys the association of fibrate application and the risk of following AMD in patients with dyslipidemia-related diseases. Moreover, treatment compliance is always an issue for chronic diseases, including dyslipidemia, in which a poor compliance in patients with dyslipidemia would lead to the development of coronary heart disease [15], but whether such compliance influences the risk of other dyslipidemia-related complication like AMD development still need further elucidation.

Herein, we aimed to evaluate the correlation between the use of fibrate medications and the subsequent AMD in patients with dyslipidemia-related diseases via the National Health Insurance Research Database (NHIRD) of Taiwan. In addition, a subgroup analysis of the medical treatment compliance of fibrate was also conducted in the multivariable analysis.

## 2. Materials and Methods

### 2.1. Data Source

This retrospective population-based cohort study adhered to the Declaration of Helsinki in 1964 and its later amendments and was approved by both the National Health Insurance Administration and the Institutional Review Board of Chung Shan Medical University Hospital (Project identification code: CSMUH CS2-15061, date of approval: 1 June 2015). Besides, the need for informed consent was also waived by the above institutions. The total population of Taiwan is around 22 to 23 million and the NHIRD contains data of insurance claims from nearly all the Taiwan’s inhabitants, and the Longitudinal Health Insurance Database, which is a derivative from NHIRD that also produced by the National Health Insurance Administration, was used in the current study. In more detail, around 1,000,000 individuals were randomly selected from the NHIRD in the year of 2005 and chosen from the Longitudinal Health Insurance Database, which can be regarded as a reduced version of NHIRD. The reason that the National Health Insurance Administration selected patients from at the years of 2005 to constitute the Longitudinal Health Insurance Database is to ensure the patient enrolled in the research has sufficient past medical history (up to five years) and an adequate follow up period (up to eight years). Then the medical data of each subject was traced from 1 January 2000 to 31 December 2013. The International Classification of Diseases Ninth Revision (ICD-9) was used for disease diagnosis in the current study. Besides, the demographic data, residence, socioeconomic status, laboratory exam and medicine prescribed by physician were all available in the Longitudinal Health Insurance Database and NHIRD. The medical prescription in Taiwan or in the NHIRD means that the physician prescribed certain medicine to patient and the patient obtain the medicine at hospital, clinic or pharmacy via the prescription note gave by the physician. The acquirement of over-the-counter medicine is not included as the “medical prescription:” in NHIRD.

### 2.2. Patient Selection

Patients were defined as having dyslipidemia-related diseases if the following criteria were fulfilled: (1) a diagnosis of chronic ischemic heart disease, atherosclerosis or hyperlipidemia from 2000 to 2012 according to corresponding ICD-9 diagnostic codes; (2) the receipt of any blood lipid profile examination before or at the same time of dyslipidemia-related diseases diagnosis; and (3) the dyslipidemia-related diseases were diagnosed by an internal medicine physician. The index date was set as one year after the diagnosis of dyslipidemia-related diseases. In addition, to exclude individuals with extremely severe health impairment, the following exclusion criteria were applied in the current study: (1) had admitted to intensive care unit before the index date, (2) had received coronary artery bypass graft surgery or percutaneous coronary intervention before the index date (3) the receipt of a diagnosis of legal blindness before the index date, (4) the receipt of a diagnosis of ocular tumors at any times, (5) the receipt of ocular removal surgery before the index date, (6) the development of AMD before the index date, (7) aged lesser than 20 or more than 100 years old at the time of the diagnosis of dyslipidemia-related diseases, (8) died before index date, and (9) had missing demographic data. In the next step, the study population was divided to those received fibrate therapy and those without such medication, then the patients received fibrate treatment were propensity-score matched to a dyslipidemia patients without fibrate use with a 1:2 ratio and caliper of 0.01 to constitute the study and control group. In the subgroup analysis, patients in the both group were divided into different subgroups according to age, gender, use of other systemic medications and the MPR of fibrate analyzed in the statistical model.

### 2.3. Main Outcome Measurement

The primary outcome of the current study is the development of AMD which is according to (1) the receipt of a diagnosis of AMD; (2) the arrangement of optical coherence tomography before or at the time of AMD diagnosis; and (3) the AMD diagnosis was made by an ophthalmologist. Moreover, the incidences of dry-AMD and neovascular AMD (nAMD) were analyzed separately to find the effect of fibrate on different AMD subtypes. The definition of nAMD was based on the coexistence of ICD-9 diagnostic codes of AMD and the use of anti-vascular endothelial growth factor therapy according to the related ATC codes since the anti-vascular endothelial growth factor therapy is always used to manage nAMD [16].

### 2.4. Demographic Variables and Co-Morbidities

To make the general status of study and control groups more comparable and reduce bias, we evaluated the influence of age, gender, residence, occupation and the following systemic/ocular co-morbidities on the development of AMD in the multivariable analysis [17,18,19]: hypertension, diabetes mellitus (DM), cerebrovascular disease, dementia, hemiplegia or paraplegia, cataract, glaucoma and optic neuropathy. Besides, the protective effects of the following medications on the risk of AMD were also considered in the analysis [13,20,21]: statin, aspirin, metformin, angiotensin-converting enzyme inhibitors (ACEi), angiotensin receptor blockers (ARB), calcium channel blocker (CCB) and beta-blocker. The presences of above medications were based on the emergence of related ATC codes. Because most individuals in the Taiwan are Han Taiwanese, ethnicity was not considered as a covariate. We longitudinally traced the data from the index date until the date of AMD diagnosis, patient withdrawal from the National Health Insurance program, or the end of NHIRD which means 31 December 2013.

### 2.5. Statistical Analyses

**Figure 1:**
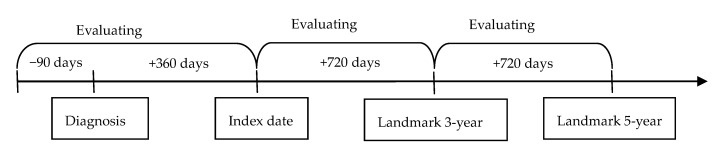
The definition of landmark time point.

SAS version 9.4 (SAS Institute Inc., Cary, NC, USA) was employed for all the analyses in the current study. First, the fibrate population and non-fibrate users were matched by propensity score matching which corporate the demographic data, co-morbidities and systemic medications, and the homogeneity of one parameters between the two groups was defined as an absolute standardized difference (ASD) lesser than 0.1. The descriptive analysis was used for the presence of basic characters. Then the incidence rate and related 95% confidence intervals (CI) of any AMD, dry-AMD and nAMD were calculated via the use of Poisson regression. After that, the Cox proportional hazard regression was used to produce the adjusted hazard ratios (aHR) of any AMD, dry-AMD and nAMD by incorporating the preceding demographic data, co-morbidities and systemic medications. Also, the Kaplan–Meier curve was drawn to demonstrate the cumulative incidence proportion of AMD between the study and control groups. For the sensitivity analysis, patients in the study and control groups were analyzed by the difference of age, gender and the use of other systemic medications. Furthermore, the MPR of fibrate was calculated which had been used as an index of medical compliance previously [22]. We conducted the landmark analysis to survey whether the MPR of fibrate would influence the development of AMD. Except the initial index date, there were two more landmark points after the index date which means three and five years after the diagnosis of dyslipidemia-related diseases, and the landmark analysis only included the patients who were at risk at the landmark time point (Figure 1). Moreover, the MPR in the landmark analysis was divided into the baseline MPR, past MPR and current MPR based on the date of landmark time point. The MPR was categorized into lesser than five percent, five to ten percent and more than ten percent in the past and current analysis, while the category of baseline MPR is ranged from five percent to more than 20 percent. Then the interaction of MPR degree and MPR time status on the risk of AMD was evaluated and the details were shown in Table 1. Statistical significance was set at *p* < 0.05. A *p* value lesser than 0.0001 was depicted as *p* < 0.0001.

## 3. Results

**Figure 2:**
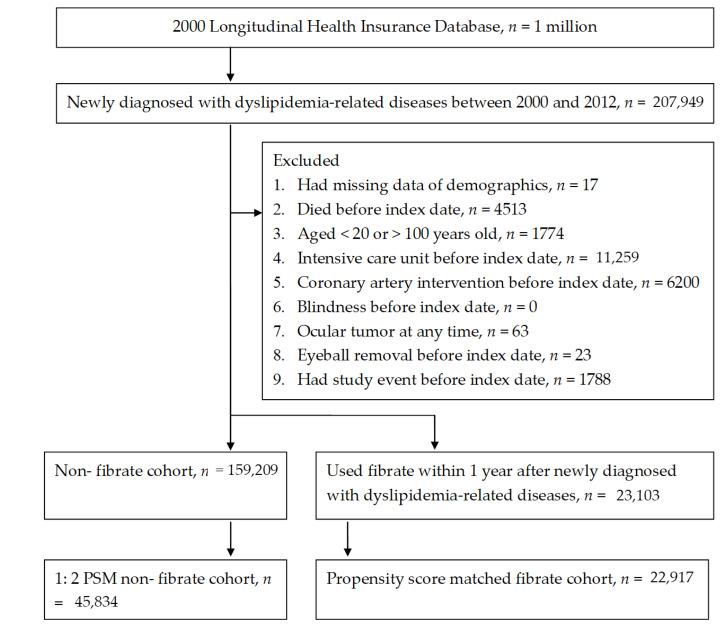
Flowchart of patient selection.

After selection, a total of 22,917 dyslipidemia patients and 45,834 non-dyslipidemia individuals were enrolled in the study and control group respectively, and the flow chart of subject selection is shown in Figure 2. The distributions of basic characteristics including index date, age, gender, residence, occupation, co-morbidities and systemic medications were similar between the two groups due to the propensity score matching process (Table 2).

**Figure 3:**
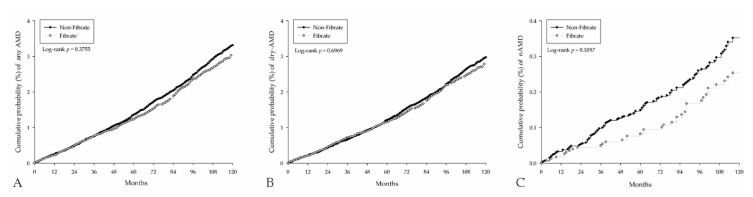
The Kaplan-Meier curves for the incidence of age-related macular degeneration. (**A**) Incidence of any types of age-related macular degeneration. (**B**) Incidence of dry age-related macular degeneration. (**C**) Incidence of neovascular age-related macular degeneration.

The median of follow-up intervals were 99 months and 98 months in the study and control groups, respectively (Table 3). There were 572 and 1181 events of any AMD development in the study and control groups which showed identical risk of AMD (aHR: 0.94, 95% CI: 0.85–1.04), and same phenomenon were observed about the risk of dry-AMD (aHR: 0.96, 95% CI: 0.86–1.06) and nAMD (aHR: 0.78, 95% CI: 0.56–1.08) (Table 3). Besides, the cumulative probability of AMD between the two groups also revealed an insignificant difference (Figure 3). On the other hand, the covariates that related to the increased occurrence of any types of AMD included the age older than 40 years old (all *p* < 0.05), male gender (*p* = 0.0469), DM (*p* = 0.0403), cataract (*p* < 0.0001), and optic neuropathy (*p* < 0.0001). The use of any medications other than fibrate did not alter the chance of AMD development (all *p* > 0.05) (Table 4).

In the sensitivity analysis, there were no significant differences concerning any AMD, dry-AMD and nAMD in dyslipidemia-related patients with different age, gender or the used of other systemic medications (Table 5). For the landmark analysis, however, a significant reduced risk of any AMD was found in patients reached a baseline MPR more than 20% (aHR: 0.729, 95% CI: 0.599–0.887, *p* = 0.0016) and past MPR more than 5% plus current MPR more than 10% three years after the diagnosis of dyslipidemia-related diseases (aHR: 0.712, 95% CI: 0.557–0.909, *p* = 0.0065) compared to those with low MPR at that time (Table 6). In addition, a significantly lower risk of dry-AMD was also found in patients with the above conditions (aHR: 0.736, 95% CI: 0.599–0.906, *p* = 0.0038 and aHR: 0.721, 95% CI: 0.557–0.934, *p* = 0.0133, respectively) (Table 6). Still, neither the landmark time point nor the MPR of fibrate would lead to prominent influence for nAMD development (all *p* > 0.05) (Table 6).

## 4. Discussion

In the current study, the use of fibrate will lead to protective effect on the development of AMD if a baseline MPR more than 20% and a persistent MPR more than 5% three years after the diagnosis of dyslipidemia-related diseases. Moreover, the condition is mainly due to the decreased incidence of dry-AMD. The factors that lead to the occurrence of AMD in dyslipidemia-related diseases include older age, male gender, systemic disease of DM, cataract, and optic neuropathy.

There were some evidences to propose the relationship between dyslipidemia and AMD [10,11]. The pathophysiology of AMD is multifactorial with including the neutral lipids as well as proteins precipitate processes, choroid angiogenesis and the inflammatory processes with the complementary system involvement [9,23]. In a previous experimental study, the component of basal linear deposit and subretinal drusenoid deposit in AMD contain the apolipoprotein B,E-containing, cholesterol-rich lipoproteins [24], and the hyperlipidemia may accelerate this process and lead to the occurrence of AMD. Moreover, the lipid material in drusen showed similar propensity with the atherosclerotic plaques. [25]. On the other hand, the hyperlipidemia status would lead to the inflammatory reaction in which the adipokines would trigger a low-grade chronic inflammation status via activating the secretion of monocyte chemotactic protein, tumor necrosis factor, and interleukin [26]. Also, hyperlipidemia is frequently prevalent in rheumatoid arthritis and related to the disease process [27]. Since the presence of dyslipidemia may trigger the development of both the dry AMD and the nAMD, the control of dyslipidemia via statins, fibrate or other lipid-lowering medications may have the potential to reduce the development of subsequent dyslipidemia-related complications like AMD in the dyslipidemia population. Moreover, medical treatment compliance is always an issue for the treatment outcome, in which a lower-than-expectation effect of statin on the decrement of cholesterol level was found due to the poor patient compliance [28]. Consequently, we speculate that the medical compliance of lipid-lower medication like fibrate, not only the prescription of fibrate, would influence the incidence of subsequent dyslipidemia-related complication such as AMD. This hypothesis is partially supported by the analysis of the current study.

The protective effect of statin of the retardation of AMD incidence has been confirmed before [12,13], while there is little evidence for the impact of fibrate on AMD development. In the current study, the application of fibrate medications did not lead to the decrement of AMD incidence though a lower aHR was found, but if we survey the relationship between MPR and the occurrence of AMD, a significant reduction of AMD incidence were observed in those with better MPR in the time of initial diagnosis and the time three years later than the initial diagnosis. To our knowledge, this is the first preliminary daa that reveals a negative association of fibrate with good MPR and AMD. Moreover, we considered a number of protective and risk factors, including the possible protective effect of statins, in the multivariable model thus the fibrate is more likely an independent protective factor for AMD. This concept is further strengthened by the sensitivity analysis regarding the applications of other medications that the aHR of AMD did not differ in fibrate patients no matter whether other medications were used. In short, we propose that the risk of AMD may be reduced in patients who use fibrate with good MPR.

In the sensitivity analysis according to the subtype of AMD, the dry-AMD showed a significantly lower incidence in patients that received fibrate with a relative higher MPR. This phenomenon is reasonable since the lipid would directly contribute to the formation of drusen and the subsequent AMD [24]. In addition, the calcified mineral in drusen-like hydroxyapatite is correlated to the formation of atherosclerotic plaques [25]. On the contrary, the incidence of nAMD did not show any difference, regardless of both the use of fibrate and the MPR of fibrate. It is possible that the influence of systemic inflammation induced by the dyslipidemia status is not strong enough to increase the chance of nAMD. Also, the nAMD has a generally lower incidence than dry-AMD which account for approximately 15% of all the AMD population [29], and the distribution of nAMD in the current study is similar to that number. Accordingly, the relatively lower number of nAMD in the current study may lead to some statistical bias. Still, the lower aHR of the nAMD in patients under fibrate use might indicate a potential protective effect for which a study with a longer follow up period should be conducted.

As the lifestyle of Western countries has spread across the globe, dyslipidemia and the related metabolic syndrome have revealed a trend to increase [30,31]. In recent nationwide studies, the prevalence of dyslipidemia was up to 34% in the Chinese population [32]. On the other hand, the AMD also influences a huge number of individuals, among which about 1.8 million patients in the United States have either dry-AMD or nAMD [33]. Moreover, AMD is the major cause of legal blindness after only cataract and glaucoma and influences various aspects of life [34,35]. Since the influences of both the dyslipidemia and AMD on human health are prominent, it should be stated that the regular use of fibrate could prevent potential AMD and blindness in patients suffering from dyslipidemia-related disorders.

The risk factors for the development of AMD in the current study include the older age, male gender, DM, cataract as well as optic neuropathy. Age is a well-established risk factor for AMD and the findings of the current study correlate to the previous concepts [9]. Regarding ocular disorders and AMD, the prominent association between cataract and AMD in the current study may be because both diseases tend to occur in the elderly and such correlation was also found in previous study [17]. Besides, the optic neuropathy is related to neuron damage [36], and AMD is also related to neurosensory cell death [23]. Accordingly, the positive correlation between the two diseases may indicate that the nervous systems in individuals with both diseases are more vulnerable. On the other hand, the mechanism of positive correlation of DM as well as male gender to AMD development is not clear and previous studies showed inconclusive results [37,38], thus further study may be needed. Interestingly, the use of statins did not lower the risk of AMD in the current study which is not in agreement with previous experience [12,13,14], but there were also some studies that showed an insignificant effect of statin on AMD prevention [39,40]. Maybe MPR also plays a role in the relationship between statins and AMD occurrence.

Several limitations remain in the current study. Firstly, its retrospective nature would lead to some bias, even after the matching process and multivariable analysis. Second, the use of insurance claim data leaves some important information unavailable such as the blood lipid levels, the severity of AMD, the clinical images of AMD and the laterality of AMD. Besides, the MPR only presents the medications that physicians prescribe to patients but not the medications they actually use, thus it cannot exactly represent patient compliance [22]. Finally, we enrolled patients with hyperlipidemia, atherosclerosis and chronic ischemic heart disease as the dyslipidemia populations in which the three diseases have different pathophysiology. Still, since all three diseases are related to impaired blood lipid levels [1,41], their enrollment as populations with lipid disorders might be considered reasonable.

## 5. Conclusions

In conclusion, the application of fibrate in patients with dyslipidemia will decrease the risk of the AMD development after considering multiple covariates. Furthermore, this benefit is seen only in individuals with good medical treatment compliance in the initial three years of dyslipidemia diagnosis. Consequently, patients with dyslipidemia should be informed that adherence to physicians’ orders to use fibrate is not only for the blood lipid control, but also for the prevention of visual impairment. Further large scale prospective studies to investigate whether the degree of dyslipidemia would influence the treatment difficulty of AMD are mandatory.

## Figures and Tables

**Table 1:** The details of subgroup in landmark analysis of medical procession ratio.

Group	Landmark Point 1 (+1080 Days after Diagnosis)		Landmark Point 2 (+1800 Days after Diagnosis)	
	Past Use (−90 to +720 Days from Diagnosis)	Current Use (+720 to +1080 Days)	Past Use (−90 to +1440 Days)	Current Use (+1440 to +1800 Days)
1	<5%	<10%	<5%	<10%
2	<5%	≥10%	<5%	≥10%
3	≥5%	≥10%	≥5%	≥10%
4	≥5%	≥10%	≥5%	≥10%

**Table 2:** Characteristics among fibrate and non-fibrate group after propensity score matching.

Characteristics	Non-Fibrate	Fibrate	ASD
N	45,834	22,917	
Year of index			0.000
2000–2003	19,918 (43.46%)	9963 (43.47%)	
2004–2007	13,246 (28.90%)	6650 (29.02%)	
2008–2012	12,670 (27.64%)	6304 (27.51%)	
Age			0.040
20–30	874 (1.91%)	464 (2.02%)	
30–40	4962 (10.83%)	2612 (11.40%)	
40–50	12,085 (26.37%)	6151 (26.84%)	
50–60	13,662 (29.81%)	6581 (28.72%)	
60–70	8815 (19.23%)	4323 (18.86%)	
70–80	4525 (9.87%)	2314 (10.10%)	
80–100	911 (1.99%)	472 (2.06%)	
Gender			0.008
Female	18,456 (40.27%)	9609 (41.93%)	
Male	27,378 (59.73%)	13,308 (58.07%)	
Urbanization			0.033
Urban	27,271 (59.50%)	13,653 (59.58%)	
Sub-urban	13,765 (30.03%)	6814 (29.73%)	
Rural	4798 (10.47%)	2450 (10.69%)	
Occupation			0.016
Government employees	2798 (6.10%)	1495 (6.52%)	
Labor	27,150 (59.24%)	13,353 (58.27%)	
Farmer and fisherman	8690 (18.96%)	4289 (18.72%)	
Low income	223 (0.49%)	132 (0.58%)	
Unemployed	6443 (14.06%)	3344 (14.59%)	
Others	530 (1.16%)	304 (1.33%)	
Co-morbidities			
Hypertension	23,090 (50.38%)	11,733 (51.20%)	0.016
DM	15,761 (34.39%)	8055 (35.15%)	0.016
Cerebrovascular disease	3488 (7.61%)	1873 (8.17%)	0.021
Dementia	119 (0.26%)	64 (0.28%)	0.004
Hemiplegia or paraplegia	555 (1.21%)	305 (1.33%)	0.011
Cataract	3960 (8.64%)	2088 (9.11%)	0.017
Glaucoma	845 (1.84%)	480 (2.09%)	0.018
Optic neuropathy	51 (0.11%)	32 (0.14%)	0.008
Medications			
Statin	8439 (18.41%)	4341 (18.94%)	0.014
Aspirin	9431 (20.58%)	4916 (21.45%)	0.021
Metformin	9463 (20.65%)	4778 (20.85%)	0.005
ACEi	7735 (16.88%)	4009 (17.49%)	0.016
ARB	5012 (10.94%)	2665 (11.63%)	0.022
CCB	14,138 (30.85%)	7245 (31.61%)	0.017
Beta-blocker	12,554 (27.39%)	6533 (28.51%)	0.025

N: number; ASD: absolute standardized difference; DM: diabetes mellitus; ACEi: angiotensin-converting enzyme inhibitors; ARB: angiotensin receptor blockers; CCB: calcium channel blocker.

**Table 3:** Study event in study groups.

Event	Non-Fibrate	Fibrate
N	45,834	22,917
Median follow up months (IQR)	98 (55–136)	99 (56–136)
Observed person-months	4,310,080	2,180,231
Any AMD		
Event of AMD	1181	572
Incidence rate * (95% CI)	2.74 (2.59–2.90)	2.62 (2.42–2.85)
Crude hazard ratio (95% CI)	Reference	0.96 (0.87–1.06)
aHR (95% CI)	Reference	0.94 (0.85–1.04)
Dry-AMD		
Event of AMD	1052	522
Incidence rate * (95% CI)	2.44 (2.30–2.59)	2.39 (2.20–2.61)
Crude hazard ratio (95% CI)	Reference	0.98 (0.88–1.09)
aHR (95% CI)	Reference	0.96 (0.86–1.06)
nAMD		
Event of AMD	129	50
Incidence rate * (95% CI)	0.30 (0.25–0.36)	0.23 (0.17–0.30)
Crude hazard ratio (95% CI)	Reference	0.76 (0.55–1.06)
aHR (95% CI)	Reference	0.78 (0.56–1.08)

N: number; IQR: interquartile range; AMD: age-related macular degeneration; nAMD: neovascular age-related macular degeneration; CI: confidence intervals; aHR: adjusted hazard ratios, co-variates including demographic data, co-morbidities and medications; * Crude incidence rate, per 10,000 person months.

**Table 4:** Cox regression for estimate the hazard ratio of age-related macular degeneration.

Covariates	aHR (95% CI)	*p* Value
Fibrate	0.935 (0.846–1.034)	0.1925 *
Year of index (ref = 2000–2003)		
2004–2007	0.817 (0.722–0.925)	0.0014 *
2008–2012	0.900 (0.744–1.090)	0.2826
Age (reference = 30–40)		
20–30	0.187 (0.026–1.370)	0.0989
40–50	1.622 (1.108–2.374)	0.0128
50–60	4.771 (3.341–6.813)	<0.0001 *
60–70	8.680 (6.075–12.402)	<0.0001 *
70–80	12.530 (8.702–18.041)	<0.0001 *
80–100	11.030 (7.098–17.141)	<0.0001 *
Gender (reference = Female)		
Male	1.102 (1.001–1.214)	0.0469
Urbanization (reference = Urban)		
Sub-urban	1.026 (0.914–1.151)	0.6651
Rural	0.927 (0.772–1.114)	0.4206
Occupation (reference = Labor)		
Government employees	1.027 (0.852–1.237)	0.7815
Farmer and fisherman	0.943 (0.814–1.094)	0.4396
Low income	1.102 (0.548–2.215)	0.7861
Unemployed	1.062 (0.928–1.215)	0.3812
Others	0.839 (0.532–1.323)	0.4499
Co-morbidities		
Hypertension	1.067 (0.940–1.212)	0.3147
DM	1.142 (1.006–1.297)	0.0403 *
Cerebrovascular disease	0.948 (0.811–1.108)	0.5026
Dementia	1.242 (0.617–2.502)	0.5438
Hemiplegia or paraplegia	0.884 (0.585–1.336)	0.5597
Cataract	1.582 (1.404–1.781)	<0.0001 *
Glaucoma	1.239 (0.968–1.586)	0.0888
Optic neuropathy	3.560 (1.945–6.515)	<0.0001 *
Medications		
Statin	1.028 (0.907–1.165)	0.6691
Aspirin	1.085 (0.972–1.212)	0.1465
Metformin	1.125 (0.973–1.301)	0.1122
ACEi	0.997 (0.884–1.125)	0.9617
ARB	0.977 (0.828–1.153)	0.7830
CCB	1.039 (0.925–1.166)	0.5200
Beta-blocker	0.974 (0.873–1.087)	0.6420

aHR: adjusted hazard ratios, covariates including demographic data, co-morbidities and medications; CI: confidence intervals; DM: diabetes mellitus; ACEi: angiotensin-converting enzyme inhibitors; ARB: angiotensin receptor blockers; CCB: calcium channel blocker; *denotes significant correlation to the development of age-related macular degeneration.

**Table 5:** Sensitivity analysis for the incidence of age-related macular degeneration.

Subgroup	aHR (95% CI)		
	Any AMD	Dry AMD	nAMD
Age (year)			
20–50	0.823 (0.611–1.107)	0.796 (0.576–1.100)	0.984 (0.464–2.087)
50–70	0.962 (0.858–1.079)	0.974 (0.863–1.098)	0.856 (0.588–1.244)
70–100	1.035 (0.884–1.212)	1.080 (0.917–1.270)	0.592 (0.300–1.167)
Gender			
Male	0.964 (0.846–1.098)	0.983 (0.856–1.129)	0.827 (0.558–1.227)
Female	0.982 (0.871–1.108)	1.001 (0.884–1.135)	0.783 (0.496–1.234)
Other medication			
With	1.003 (0.908–1.108)	1.032 (0.930–1.145)	0.746 (0.528–1.056)
Without	0.847 (0.698–1.028)	0.833 (0.678–1.022)	0.975 (0.548–1.737)

aHR: adjusted hazard ratios, covariates including demographic data, co-morbidities and medications; CI: confidence intervals; AMD: age-related macular degeneration; nAMD: neovascular age-related macular degeneration.

**Table 6:** Time varying and dosage of fibrate on risk of any AMD.

MPR	Any AMD		Dry AMD		nAMD	
	aHR (95% CI)	*p* Value	aHR (95% CI)	*p* Value	aHR (95% CI)	*p* Value
At baseline (+1 year from diagnosis)						
0%	Reference		Reference		Reference	
<5%	0.960 (0.823–1.120)	0.6067	0.974 (0.829–1.145)	0.7512	0.841 (0.506–1.399)	0.5048
5–10%	1.013 (0.847–1.213)	0.8864	1.039 (0.861–1.254)	0.6863	0.794 (0.429–1.471)	0.4638
10–20%	1.060 (0.888–1.265)	0.5175	1.092 (0.908–1.314)	0.3502	0.791 (0.427–1.466)	0.4569
≥20%	0.729 (0.599–0.887)	0.0016 *	0.736 (0.599–0.906)	0.0038 *	0.672 (0.362–1.248)	0.2087
Landmark 1 (+3 years from diagnosis)						
Past < 5%, Current < 10%	Reference		Reference		Reference	
Past < 5%, Current ≥ 10%	0.961 (0.676–1.367)	0.8254	0.985 (0.680–1.426)	0.9344	0.777 (0.246–2.449)	0.6663
Past ≥ 5%, Current < 10%	1.038 (0.896–1.203)	0.6162	1.093 (0.938–1.273)	0.2557	0.588 (0.331–1.043)	0.0692
Past ≥ 5%, Current ≥ 10%	0.712 (0.557–0.909)	0.0065 *	0.721 (0.557–0.934)	0.0133 *	0.633 (0.294–1.364)	0.2430
Landmark 2 (+5 years from diagnosis)						
Past < 5%, Current < 10%	Reference		Reference		Reference	
Past < 5%, Current ≥ 10%	1.042 (0.735–1.476)	0.8187	1.131 (0.793–1.612)	0.4966	0.292 (0.041–2.096)	0.2208
Past ≥ 5%, Current < 10%	0.883 (0.731–1.066)	0.1949	0.902 (0.740–1.098)	0.3041	0.732 (0.391–1.373)	0.3316
Past ≥ 5%, Current ≥ 10%	0.860 (0.664–1.112)	0.2502	0.906 (0.694–1.183)	0.4679	0.489 (0.179–1.338)	0.1638

MPR: medical possession ratio; aHR: adjusted hazard ratios, covariates including demographic data, co-morbidities and medications; CI: confidence intervals; AMD: age-related macular degeneration; nAMD: neovascular age-related macular degeneration; * denotes significant correlation to the development of age-related macular degeneration.

## Data Availability

Not applicable.

## References

[1] Durrington P. (2003). Dyslipidaemia. Lancet.

[2] Ikeda N., Nishi N., Sugiyama T., Noda H., Noda M. (2020). Effective coverage of medical treatment for hypertension, diabetes and dyslipidaemia in japan: An analysis of national health and nutrition surveys 2003-2017. J. Health Serv. Res. Policy.

[3] Gonzalez L., Helkin A., Gahtan V. (2016). Dyslipidemia part 2: Review of dyslipidemia treatment in patients with noncoronary vascular disease. Vasc. Endovascular. Surg..

[4] Kopin L., Lowenstein C. (2017). Dyslipidemia. Ann. Intern. Med..

[5] Rathnakumar K., Ramachandran K., Baba D., Ramesh V., Anebaracy V., Vidhya R., Vinothkumar R., Poovitha R., Geetha R. (2018). Prevalence of dry eye disease and its association with dyslipidemia. J. Basic Clin. Physiol. Pharmacol..

[6] Fu Z., Chen C.T., Cagnone G., Heckel E., Sun Y., Cakir B., Tomita Y., Huang S., Li Q., Britton W. (2019). Dyslipidemia in retinal metabolic disorders. EMBO Mol. Med..

[7] Yau J.W., Lee P., Wong T.Y., Best J., Jenkins A. (2008). Retinal vein occlusion: An approach to diagnosis, systemic risk factors and management. Intern. Med. J..

[8] Luo R.J., Liu S.R., Li X.M., Zhuo Y.H., Tian Z. (2010). Fifty-eight cases of ocular ischemic diseases caused by carotid artery stenosis. Chin. Med. J..

[9] Mitchell P., Liew G., Gopinath B., Wong T.Y. (2018). Age-related macular degeneration. Lancet.

[10] Shen J., He J., Wang F. (2016). Association of lipids with age-related macular degeneration. Discov. Med..

[11] Ghaem Maralani H., Tai B.C., Wong T.Y., Tai E.S., Li J., Wang J.J., Mitchell P. (2015). Metabolic syndrome and risk of age-related macular degeneration. Retina.

[12] Ma L., Wang Y., Du J., Wang M., Zhang R., Fu Y. (2015). The association between statin use and risk of age-related macular degeneration. Sci. Rep..

[13] Lee H., Jeon H.L., Park S.J., Shin J.Y. (2019). Effect of statins, metformin, angiotensin-converting enzyme inhibitors, and angiotensin ii receptor blockers on age-related macular degeneration. Yonsei Med. J..

[14] Vavvas D.G., Daniels A.B., Kapsala Z.G., Goldfarb J.W., Ganotakis E., Loewenstein J.I., Young L.H., Gragoudas E.S., Eliott D., Kim I.K. (2016). Regression of some high-risk features of age-related macular degeneration (amd) in patients receiving intensive statin treatment. EBioMedicine.

[15] Valdez C.A., Ulrich H. (2005). Similar medication compliance and control of dyslipidemia with simvastatin or atorvastatin in a staff-model hmo medical clinic. J. Manag. Care Pharm..

[16] Supuran C.T. (2019). Agents for the prevention and treatment of age-related macular degeneration and macular edema: A literature and patent review. Expert Opin. Ther. Pat..

[17] Vassilev Z.P., Ruigómez A., Soriano-Gabarró M., García Rodríguez L.A. (2015). Diabetes, cardiovascular morbidity, and risk of age-related macular degeneration in a primary care population. Invest. Ophthalmol. Vis. Sci..

[18] Lazreg S., Delcourt C., Zeggane S., Sanchez A., Ziani A., Daghbouche M., Benmoussa S., Mokrani K., Billah Mekki M., Renault D. (2016). Age-related macular degeneration and its risk factors in north africans living in algeria and italy. Ophthalmic. Res..

[19] Fraser-Bell S., Symes R., Vaze A. (2017). Hypertensive eye disease: A review. Clin. Exp. Ophthalmol..

[20] Thomas A.S., Redd T., Hwang T. (2015). Effect of systemic beta-blockers, ace inhibitors, and angiotensin receptor blockers on development of choroidal neovascularization in patients with age-related macular degeneration. Retina.

[21] Chen Y.Y., Shen Y.C., Lai Y.J., Wang C.Y., Lin K.H., Feng S.C., Liang C.Y., Wei L.C., Chou P. (2019). Association between metformin and a lower risk of age-related macular degeneration in patients with type 2 diabetes. J. Ophthalmol..

[22] Clifford S., Perez-Nieves M., Skalicky A.M., Reaney M., Coyne K.S. (2014). A systematic literature review of methodologies used to assess medication adherence in patients with diabetes. Curr. Med. Res. Opin..

[23] Kijlstra A., Berendschot T.T. (2015). Age-related macular degeneration: A complementopathy?. Ophthalmic. Res..

[24] Curcio C.A. (2018). Soft drusen in age-related macular degeneration: Biology and targeting via the oil spill strategies. Invest. Ophthalmol. Vis. Sci..

[25] Bergen A.A., Arya S., Koster C., Pilgrim M.G., Wiatrek-Moumoulidis D., van der Spek P.J., Hauck S.M., Boon C.J.F., Emri E., Stewart A.J. (2019). On the origin of proteins in human drusen: The meet, greet and stick hypothesis. Prog. Retin. Eye Res..

[26] Jung U.J., Choi M.S. (2014). Obesity and its metabolic complications: The role of adipokines and the relationship between obesity, inflammation, insulin resistance, dyslipidemia and nonalcoholic fatty liver disease. Int. J. Mol. Sci..

[27] Bag-Ozbek A., Giles J.T. (2015). Inflammation, adiposity, and atherogenic dyslipidemia in rheumatoid arthritis: Is there a paradoxical relationship?. Curr. Allergy Asthma Rep..

[28] Frolkis J.P., Pearce G.L., Nambi V., Minor S., Sprecher D.L. (2002). Statins do not meet expectations for lowering low-density lipoprotein cholesterol levels when used in clinical practice. Am. J. Med..

[29] Gehrs K.M., Anderson D.H., Johnson L.V., Hageman G.S. (2006). Age-related macular degeneration--emerging pathogenetic and therapeutic concepts. Ann. Med..

[30] Saklayen M.G. (2018). The global epidemic of the metabolic syndrome. Curr. Hypertens. Rep..

[31] Sherling D.H., Perumareddi P., Hennekens C.H. (2017). Metabolic syndrome. J. Cardiovasc. Pharmacol. Ther..

[32] Pan L., Yang Z., Wu Y., Yin R.X., Liao Y., Wang J., Gao B., Zhang L. (2016). The prevalence, awareness, treatment and control of dyslipidemia among adults in china. Atherosclerosis.

[33] Friedman D.S., O’Colmain B.J., Muñoz B., Tomany S.C., McCarty C., de Jong P.T., Nemesure B., Mitchell P., Kempen J. (2004). Prevalence of age-related macular degeneration in the united states. Arch. Ophthalmol..

[34] Velez-Montoya R., Oliver S.C., Olson J.L., Fine S.L., Quiroz-Mercado H., Mandava N. (2014). Current knowledge and trends in age-related macular degeneration: Genetics, epidemiology, and prevention. Retina.

[35] Taylor D.J., Hobby A.E., Binns A.M., Crabb D.P. (2016). How does age-related macular degeneration affect real-world visual ability and quality of life? A systematic review. BMJ Open.

[36] Levin L.A. (2018). Neuroprotection in optic neuropathy. Asia Pac. J. Ophthalmol..

[37] Wong W.L., Su X., Li X., Cheung C.M., Klein R., Cheng C.Y., Wong T.Y. (2014). Global prevalence of age-related macular degeneration and disease burden projection for 2020 and 2040: A systematic review and meta-analysis. Lancet Glob. Health.

[38] Chakravarthy U., Wong T.Y., Fletcher A., Piault E., Evans C., Zlateva G., Buggage R., Pleil A., Mitchell P. (2010). Clinical risk factors for age-related macular degeneration: A systematic review and meta-analysis. BMC Ophthalmol..

[39] Shalev V., Sror M., Goldshtein I., Kokia E., Chodick G. (2011). Statin use and the risk of age related macular degeneration in a large health organization in israel. Ophthalmic. Epidemiol..

[40] Roizenblatt M., Naranjit N., Maia M., Gehlbach P.L. (2018). The question of a role for statins in age-related macular degeneration. Int. J. Mol. Sci..

[41] Lee J.S., Chang P.Y., Zhang Y., Kizer J.R., Best L.G., Howard B.V. (2017). Triglyceride and hdl-c dyslipidemia and risks of coronary heart disease and ischemic stroke by glycemic dysregulation status: The strong heart study. Diabetes Care.

